# Skin Adverse Events During Dual and Triple Therapy for HCV-Related Cirrhosis

**DOI:** 10.5812/hepatmon.16632

**Published:** 2014-03-11

**Authors:** Alessandro Federico, Dolores Sgambato, Gaetano Cotticelli, Antonietta Gerarda Gravina, Marcello Dallio, Filippo Beneduce, Eleonora Ruocco, Marco Romano, Carmela Loguercio

**Affiliations:** 1 Department of Clinical and Experimental Medicine, Gastroenterology Unit, Second University of Naples, Naples, Italy; 2 Department of Dermatology, Second University of Naples, Naples, Italy

**Keywords:** Liver Cirrhosis, Telaprevir, Skin Diseases

## Abstract

**Introduction:**

Dermatological adverse events are an existing concern during treatment of hepatitis C virus infection. Peginterferon/ribavirin treatment is associated with well-characterized dermatological lesions tending towards a uniform entity of dermatitis. New telaprevir- or boceprevir-based triple-therapy has led to significant improvements in sustained virological response rates, although associated with an increase in cutaneous adverse events compared peginterferon/ribavirin alone.

**Case Presentation:**

We report a case of a patient who discontinued telaprevir because of severe skin eruptions and who, during ribavirin and interferon treatment, after a period free of skin lesions, developed new dermatological lesions different than those experienced during telaprevir treatment.

**Conclusions:**

Several adverse effects are associated to anti-HCV drugs, hence appropriate skin care management and follow-up are very important. A careful anamnesis before the initiation of triple therapy is necessary to identify previous dermatological diseases that could increase skin adverse effects incidence.

## 1. Introduction

Adverse cutaneous reactions are described in standard of care (SOC) therapy with peginterferon–alpha (PEG-IFN) and ribavirin (RBV) (1). These tend towards a uniform entity of dermatitis, characterized by generalized pruritus and skin xerosis, with eczematiform lesions accentuated by erythematous papules and microvesicles that are often excoriated, predominantly located on the extremities and on truncal skin sites exposed to friction. Management of these eruptions can be achieved with the same approach as for eczema (topical corticosteroids and emollients), usually without the need for discontinuation of the antiviral treatment (2).

Two protease inhibitors, boceprevir and telaprevir, recently approved for the treatment of genotype 1 HCV infection in combination with PEG-IFN and RBV have been associated with a higher sustained virological response rate than SOC therapy. During triple therapy an increased incidence of skin adverse events is described: in triple therapy phase II and III trials it has been reported a 55 % rate of cutaneous adverse events compared with 33 % in the PEG-IFN and RBV controls. Skin lesions during dual and teleprevir-containing triple therapy are similar, but the severity and frequency are significantly higher in triple therapy compared with dual therapy.

We report the case of a 64 year old man with HCV genotype 1 who discontinued telaprevir therapy for eczematiform lesions (grade 3) with improvement of the rash within seven days of telaprevir withdrawal. The patient continued PEG-IFN and RBV treatment, and, after a skin lesion-free period, showed repeated episodes of skin lesions different than those occurred during triple therapy both for type and location. The treatment was interrupted after sixth months for detectable HCV-RNA, with disappearance of skin lesions. The mechanism of these side effects is currently unclear, although these preliminary data suggest that the management of dermatological reactions will remain an important issue when dealing with anti-HCV treatment.

## 2. Case Presentation

**Figure 1.**
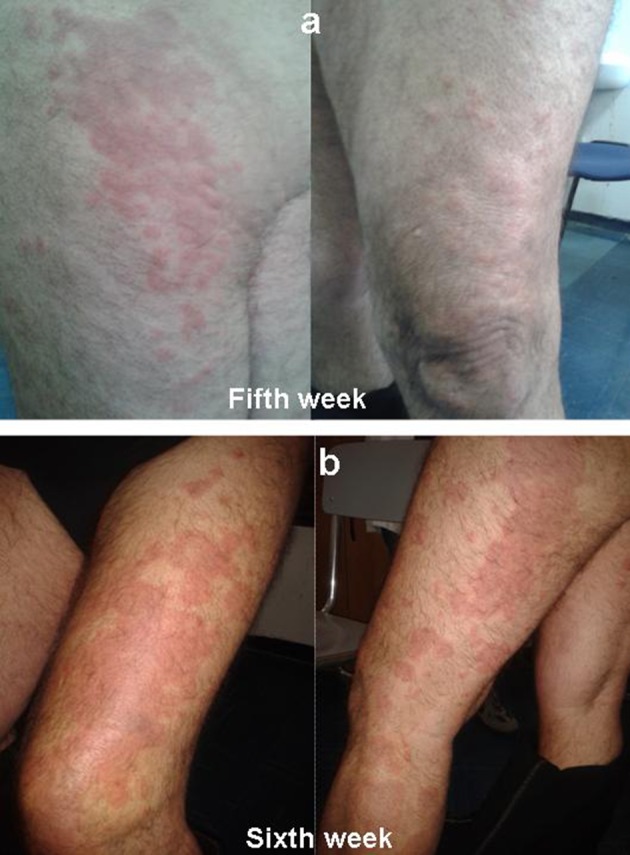
a) Eczematiform Itchy Lesion on the Thigh, Buttock, Back of the Foot, in Both Lower Limbs, Especially on the Left at Fifth Week; b) Eruption Progression to Grade 3 at Sixth Week: Rash involved More Than 50 % of Body Surface Area, Presenting Vescicles and Superficial Ulceration

A 64 year old man of Caucasian origin with HCV-related cirrhosis (fibroscan examination 19.8 kPa, F4), genotype 1b, treatment-naive, started a triple therapy including PEG-IFN α2a (180 µg/week), ribavirin (1000 mg/day) and telaprevir (2250 mg/day) in April 2013. The patient was instructed on how to prevent the occurrence of rash by limiting sun or heat exposure and avoiding tight-fitting clotting. A moisturizing cream was integrated into the treatment from the very beginning. These prophylactic measures were continued until the end of therapy. He had not reported any previous history of inflammatory skin disease. HCV-RNA was undetectable at fourth week. On fifth week, patient presented an eczematiform itchy lesion on the thigh, buttock, back of the foot, in both lower limbs, especially on the left (Figure 1A). The patient denied other symptoms (malaise, chills, skin dysesthesias, fever, cough, shortness of breath and gastrointestinal symptoms). Also, there was no evidence of lymphadenopathy. Serum test revealed anemia (hemoglobin level, 11.7 g/dL), thrombocytopenia (platelet count, 87 × 103/μL). There was no evidence of eosinophilia, neutrophilia, acute renal failure. Skin eruption involved about 36 % of Body Surface Area (BSA) (grade 1 for grading of telaprevir-associated rash severity in Phase III telaprevir trials). In accordance with guidelines, telaprevir was not discontinued (3). Administration of topical steroids and oral antihistamine drug was started. Biopsy was not performed because the feature of skin eruptions was compatible with telaprevir-related dermatological adverse events.

On sixth week, telaprevir treatment was discontinued for eruption progression to grade 3: rash involved more than 50 % BSA, presenting vesicles and superficial ulceration (Figure 1B). There was a rapidly progressing exanthema but there were not other alert signs or symptoms to suspect a severe cutaneous adverse reaction (SCAR) as Drug Reaction with Eosinophilia and Systemic Symptoms (DRESS) or Stevens Johnson Syndrome (SJS). A report to the University Hospital Pharmacovigilance Service of adverse drug reaction was issued. In accordance with guidelines, the patient continued PEG-IFN and RBV therapy, for a week, evaluating lesions evolution (4).

**Figure 2.**
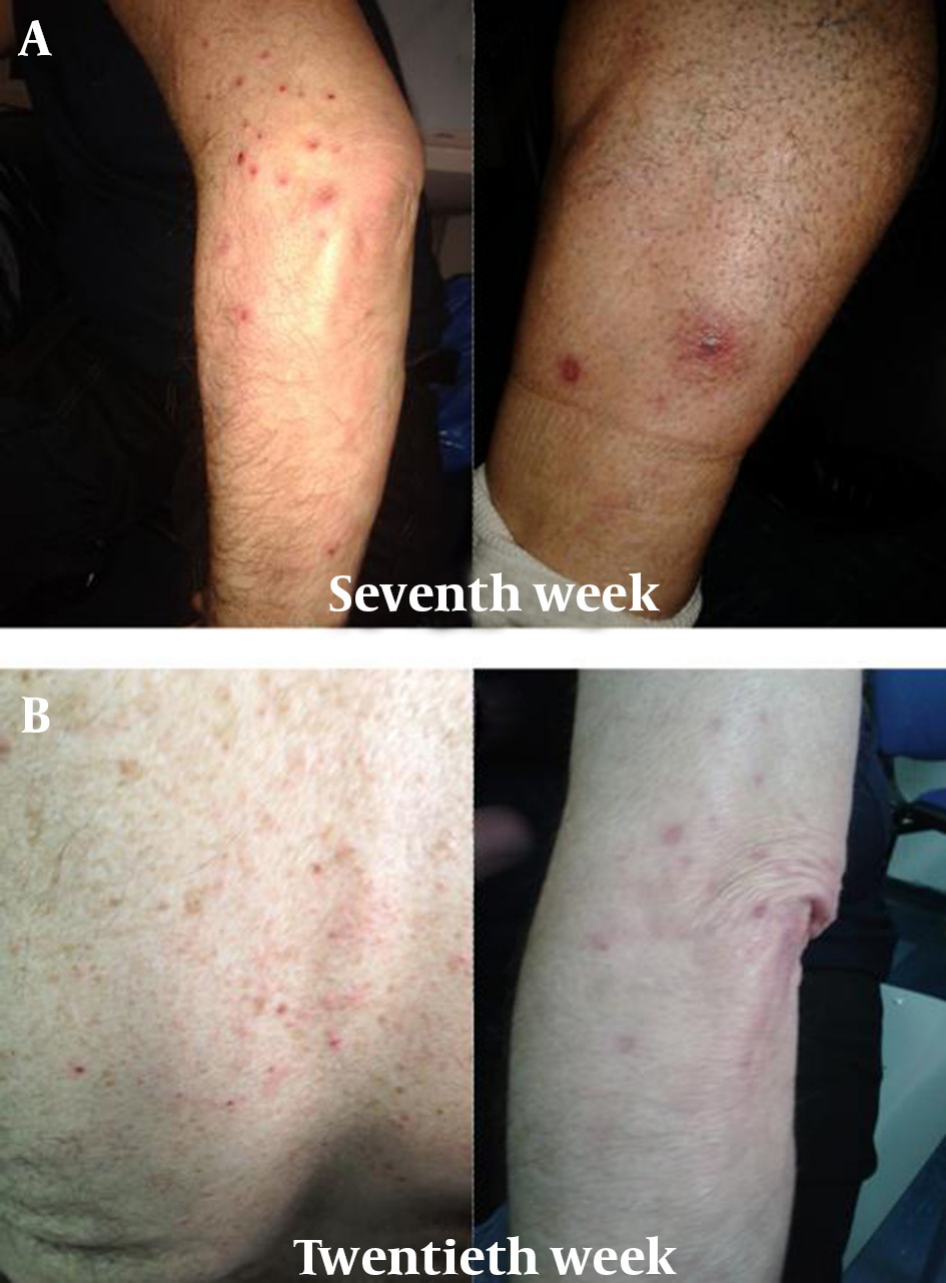
A) Localized Exanthematous Pustolosis, With Pruritus at Seventh Week; B) Pustular Lesions at the Back and Upper Limbs, Involving Less Than 50 % of Body Surface Area at Twentieth Week

After a week, skin lesions greatly improved, and, therefore, PEG-IFN and RBV were not discontinued. However, after a few days, new different dermatological lesions appeared on left lower limb. These lesions were described by the dermatologist as localized exanthematous pustolosis, with pruritus in the absence of other symptoms (Figure 2A). Because this was interpreted as a reaction compatible with RBV treatment, the dermatologist decided not to perform skin biopsy, even though this might have better clarified the nature of the skin lesion. Serum tests did was not altered. Amoxicillin (1 g table, twice daily) was administrated for ten days. The skin lesions resolved within a week, so topical steroids and antihistamine drug were stopped. HCV-RNA was undetectable at eighth and twelfth week.

The same skin manifestations recurred on the twentieth week, twelve weeks after withdrawal of telaprevir. Pustular lesions appeared at the back and upper limbs, involving less than 50 % BSA (Figure 2B). Topical steroids, antihistamine drug and amoxicillin were administered and led to prompt resolution of the skin lesions within a week.

At twenty-four weeks of treatment erythematous lesions localized on the inner part of both thighs appeared, soon after the injection of interferon. These lesions resolved spontaneously, without any treatment. At the same time, HCV-RNA became detectable, so PEG-IFN and RBV therapy was discontinued. The patient has never experienced any recurrence of dermatological lesions.

## 3. Conclusions

HCV is the major cause of chronic liver disease that gradually progresses from chronic hepatitis to cirrhosis and hepatocellular carcinoma (HCC) during the course of infection. Dermatological adverse events are described during HCV therapy. The traditional treatment PEG-IFN plus RBV combination therapy is associated with inflammatory skin lesions at the site of injection. The type of skin reactions observed are vesicle-like erythematous eruptions at the injection sites, and pruritic papular erythematous eruptions located on the face, neck, distal limbs, dorsa of-like erythematous eruptions away from the injection sites and autosensitization dermatitis apart from injection sites have not been reported frequently (5). Manjon-Haces et al described a series of 210 patients in Spain under HCV treatment with interferon alfa-2b plus ribavirin. Fourteen patients experienced localized while two had generalized eczematous lesions (6).

Dereure et al. (7) described the most extensive series with diffuse inflammatory lesions. They observed 20 patients that presented eczema-like skin lesions mainly on the extremities and sometimes associated with photosensitivity. The clinical pattern was a pruritic, confluent, papular erythematous eruption admixed with occasional vesicles. Lesions were predominantly located on the distal limbs, dorsum of the hands, face, neck and, less frequently, the trunk, axillae and buttocks. Other cutaneous side-effects at a distance from injection points include the occurrence or worsening of psoriasis, lichen planus, vitiligo, alopecia areata, lupus erythematosus, sarcoidosis, aphtae, Meyerson’s phenomenon and nummular dermatitis (8).

The introduction of the new triple-therapy including telaprevir has brought an improvement of SVR but led to an increased incidence of skin side effects in respect to SOC. More than 90 % of reports were either mild (37 % grade 1) or moderate (14 % grade 2) skin adverse events and > 90 % of cases were stable, remaining unchanged until the end of telaprevir treatment with no progression to a more severe grade. The most common presentations are characterized by pruritus, xerosis, erithematous papules, vesicles and excoriated lesions located on the trunk, extremities and friction sites (9). The pattern of lesions are similar to rash associated with PEG-IFN and RBV therapy alone but greater in frequency (55 % vs 33 %) and severity (3.7 % vs 0.4 %) (9).

A small proportion of patients (6 %) experienced more severe skin conditions. However, a few patients presented with SCAR manifestations: three cases of SJS and 11 cases of DRESS were suspected, with 2 SJS and 3 DRESS cases, but these also resolved after treatment discontinuation (9). In our case, patient was naive to HCV treatment and he denied previous history of inflammatory skin disease. During triple-therapy including telaprevir, he showed compatible cutaneous ADR which led to stopping of protease inhibitor and continuing PEG-IFN and RBV treatment. At twenty-four weeks also SOC was discontinued because HCV-RNA was detectable again. To date, the pathogenic mechanisms of telaprevir-related skin lesions are still unknown.

This case leaves a number of questions open: are the dermatological lesions appeared on the fifth only due to telaprevir or are they the consequence of PEG-IFN and RBV too? Can telaprevir administration unmask pathogenetic mechanisms that would explain cutaneous effects to PEG-IFN and RBV when stopping telaprevir? The patient would have had effects on the skin to ribavirin even if he had not been previously treated with telaprevir?

In conclusion, several adverse effects are associated to anti-HCV drugs, hence appropriate skin care management and follow-up are very important. A careful anamnesis before the initiation of triple therapy is necessary to identify previous dermatological diseases that could increase skin adverse effects incidence. Other studies are necessary to evaluate the real risk of cutaneous manifestations in HCV treatment, through the findings of clinical, hematological or genetic factors.
